# Distribution of cracks in an anchored cavern under blast load based on cohesive elements

**DOI:** 10.1038/s41598-022-08560-y

**Published:** 2022-03-16

**Authors:** Yi Luo, Chenhao Pei, Dengxing Qu, Xinping Li, Ruiqiu Ma, Hangli Gong

**Affiliations:** 1 Hubei Key Laboratory of Road-Bridge and Structure Engineering, Wuhan University of Technology, Wuhan, 430070 China; 2 School of Civil Engineering and Architecture, Wuhan University of Technology, Wuhan, 430070 China; 3 School of Safety Science and Emergency Management, Wuhan University of Technology, Wuhan, 430070 China; 4 Sanya Science and Education Innovation Park, Wuhan University of Technology, Sanya, 572000 China

**Keywords:** Civil engineering, Nonlinear phenomena

## Abstract

To explore the distribution of cracks in anchored caverns under the blast load, cohesive elements with zero thickness were employed to simulate crack propagation through numerical analysis based on a similar model test. Furthermore, the crack propagation process in anchored caverns under top explosion was analyzed. The crack propagation modes and distributions in anchored caverns with different dip angles fractures in the vault were thoroughly discussed. With the propagation of the explosive stress waves, cracks successively occur at the arch foot, the floor of the anchored caverns, and the boundary of the anchored zone of the vault. Tensile cracks are preliminarily found in rocks that surround the caverns. In the scenario of a pre-fabricated fracture in the upper part of the vault, the number of cracks at the boundary of the anchored zone of the vault first decreases then increases with the increasing dip angle of the pre-fabricated fracture. When the dip angle of the pre-fabricated fracture is 45°, the fewest cracks occur at the boundary of the anchored zone. The wing cracks deflected to the vault are formed at the tip of the pre-fabricated fracture, around which are synchronous formed tensile and shear cracks. Under top explosion, the peak displacement and the peak particle velocity in surrounding rocks of anchored caverns both reach their maximum values at the vault, successively followed by the sidewall and the floor. In addition, with the different dip angles of the pre-fabricated fracture, asymmetry could be found between the peak displacement and the peak particle velocity. The vault displacement of anchored caverns is mainly attributed to tensile cracks at the boundary of the anchored zone, which are generated due to the tensile waves reflected from the free face of the vault. When a fracture occurs in the vault, the peak displacement of the vault gradually decreases while the residual displacement increases.

## Introduction

**Figure 1:**
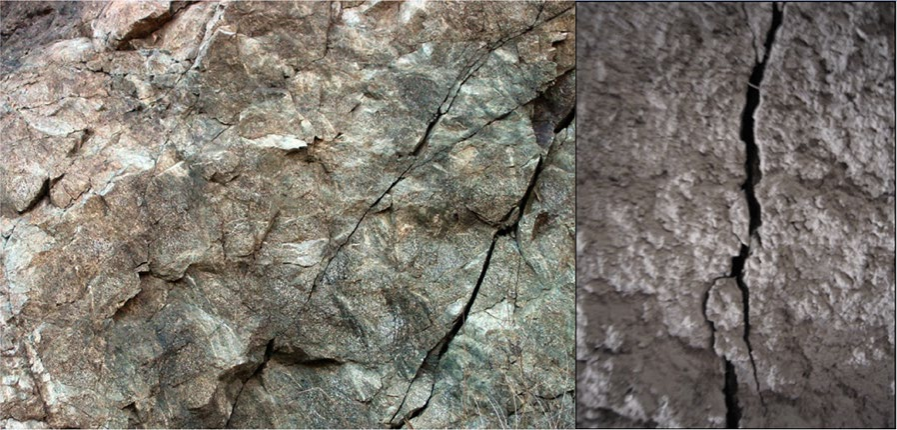
Cave rock mass fracture.

Underground engineering works are affected by dynamic loadings, such as the blast load generated during excavation of adjacent tunnels^1,2^, accidental blast load^3^, or missile attack^4^. In addition, some defects (i.e. faults and joints) are inevitably present in the rock surrounding underground caverns, as shown in Fig. 1. Fractures affect the stability of underground structures under blast load; especially, when open through-going fractures (such as faults, joints, and excavation-induced cracks) appear, not only do these fractures affect crack propagation in the vicinity of anchored caverns, but primary fractures may interact and even coalesce with other cracks in anchored caverns. Thus, it is necessary to explore crack propagation around anchored caverns with pre-existing fractures under dynamic load.

At present, research into the dynamic response of underground anchored caverns has attracted much attention. Scholars have investigated the failure modes of underground anchored caverns without pre-existing fractures. Xu et al.^5^ and Wang et al.^6^ investigated the failure of anchored caverns under blast load through physical model testing. Sivalingam et al.^7^ surveyed the stress redistribution around adjacent tunnels during blasting excavation. Xia et al.^8^ conducted field tests and numerical simulations. Li et al.^9^ analysed the influence of waves triggered by blast tunnelling with explosive charges on the peak particle velocity (PPV) and stress from adjacent tunnels. Scholars also have explored the damage and failure of underground anchored caverns with pre-existing fractures. Liu et al.^10^ investigated the propagation of fractures around tunnels under blast load through use of a tunnel model containing a spandrel crack (TMCSC) model. Zhou et al.^11^ explored the propagation characteristics of radial fractures in tunnels under the effect of impact load. Based on dynamic testing, Guo et al.^12^ investigated crack propagation in roadways with defects under blast load. As for failure characteristics of joint planes in rocks under dynamic load, Yang et al.^13^ analysed the crack propagation path, the changes in stress intensity factor, and wing-crack mechanism of different joints. Li^14^ determined certain characteristics of the interaction between stress waves and rock joints. With the aid of numerical analysis software, Deng^15^ analysed the extent, severity, and modes of failure in a circular roadway with joints under dynamic blast load.

Many scholars concentrate on the dynamic propagation of fractures or small-scale approximate tests^16–18^. The large-scale physical testing of anchored caverns under blast load is expensive and requires time. According to physical test data, the distribution and propagation of cracks in anchored caverns with pre-existing fractures were explored through the use of numerical simulation methods. Due to the discontinuity and complexity of fractures, it is difficult to simulate crack propagation using the finite element method. In the current research, cracks are approximately expressed mainly through element deletion or element damage^9,19^. Having low computational efficiency for a large-scale model, the particle flow code, discrete element, and extended finite element methods are applicable for exploring test blocks at an experimental scale^20–22^. Cohesive element has the advantages of high computational efficiency and good convergence in analyzing crack propagation^23,24^. The thickness of the cohesive element is set to zero, which is appropriate to represent crack. Based on the cohesion zone model (CZM), many scholars have studied concrete^11,25–27^ and rock^28,29^ at mesoscale, and analyzed the fracture process of rock-like materials.

The existing research on the dynamic response of anchored caverns mainly focuses on stress, displacement, and vibration velocity. There is a lack of research on the crack propagation of anchored caverns. By globally embedding cohesive elements with zero thickness, the crack propagation process in surrounding rocks of anchored caverns was analyzed; moreover, the distribution of cracks and modes of failure of anchored caverns when a pre-fabricated fracture with different dip angles was present in the vault were investigated.

## Numerical model

### Establishment of the model

**Figure 2:**
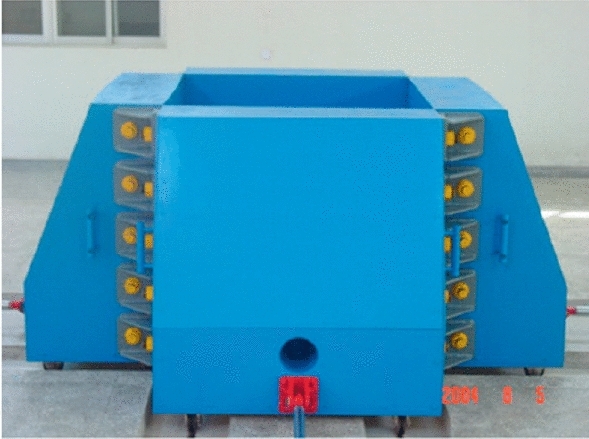
The test device^6^.

**Figure 3:**
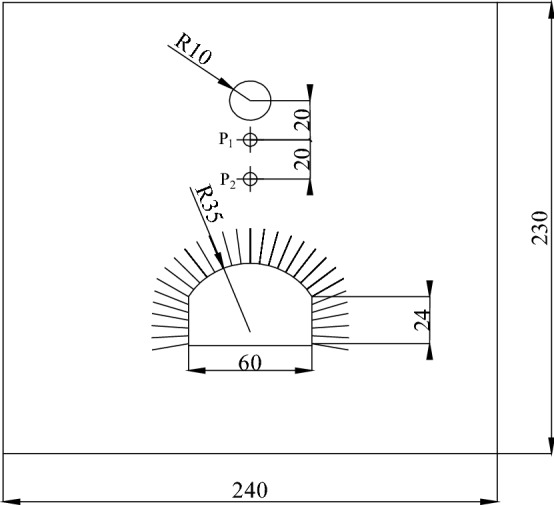
Schematic representation of the numerical model (unit: cm).

To verify the accuracy of the embedded cohesive elements with zero thickness, a numerical model was established according to the physical test model. The test equipment is shown in Fig. 2 and details are provided elsewhere^6^. The test equipment is composed of four rigid side limiting devices that can move forward and backward. On the explosion-facing surface of each side limiting device, the aluminum wave damping plate with a porosity of 50% is used to eliminate the reflection of the explosion wave on the side, so as to simulate the infinite boundary during the field test. The anti-explosion model test is mainly based on class III homogeneous surrounding rock which is often encountered in shallow underground engineering. The density scale, stress scale, and geometric scale are 0.67, 0.06, and 0.09 respectively. The model test material is mainly composed of sand, cement, water, and accelerator. The mix ratio is: *m*_sand_: *m*_cement_: *m*_water_: *m*_accelerator_ = 15:1:1.6:0.0166. In the test, an aluminum rod with a diameter of 1.84 mm is used to simulate the anchor rod. For the convenience of calculation, the physical test model was simplified into a plane strain problem. The numerical model is illustrated in Fig. 3. The model has dimensions of 2400 mm × 2300 mm × 40 mm (width × height × thickness), in which the cavern, appearing as a circular arch with vertical walls, presents a span of 60 cm and a height of 40 cm, showing the radius of the circular arch of 35 cm; the spacing between bolts, each with a length of 24 cm, is set to 4 cm. The centre of the blast source is 83 cm from the top of the cavern. At the left and right-hand boundaries and the lower part of the model, semi-infinite bodies are simulated with an infinite element.

The solid element adopts the constant strain elements, and the boundary adopts the infinite element to absorb the reflected wave. The bolt is discretized by the beam element, and the bolt deforms together with the surrounding rock. The bolts and the slippage effect thereof are simulated by the bilinear constitutive model.

The numerical simulation is very sensitive to the grid size, and the accuracy of the simulation results strongly depends on the accuracy of grid division. The size of the grid has a great influence on the explosion stress field and the stress wave propagation. According to the literature and our own trial calculations, when the number of elements in a load wavelength reaches 16, the calculated peaks and waveforms of each physical quantity almost tend to be stable. The calculation formula of wavelength is:

1 $$\lambda = c_{P} \times T$$

where c_P_ is the propagation velocity of an elastic longitudinal wave in an infinite medium, and $T$ is the loading wave period. c_P_ can be derived from the following equation.

2 $$C_{P} = \sqrt {\frac{E(1 - \mu )}{{\rho (1 + \mu )(1 - 2\mu )}}}$$

where $E$ is the elastic modulus of the material, $\mu$ is the Poisson's ratio, and $\rho$ is the material density.

According to Eqs. (1) and (2), the wavelength is 0.22 m, and the grid size is less than 13 mm. In this paper, the mesh size is 10 mm.

**Figure 4:**
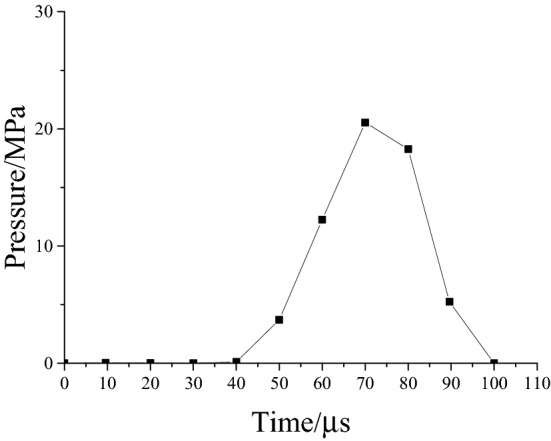
Explosion pressure versus time history curve.

TNT centralized coupling charge is used in the physical experiment. As we are interested in the crack distribution in the rock surrounding an anchored cavern, the air action near the explosion area is not considered, the blast pressure was measured by the test and applied to the blasting cavity at 10 cm from the centre of the explosive charge. The time history curve of pressure is shown in Fig. 4.

### Constitutive model

Cohesion essentially refers to the interaction between material atoms or molecules. As a simplified phenomenological model, the cohesive zone model (CZM) visualises the crack initiation and propagation. In terms of its core concept, the non-linear constitutive model of materials is described based on the relationship between cohesion and relative displacement. The bilinear CZM is applied here, for which it is supposed that materials in the cohesive zone are linear-elastic in the initial response stage; thereafter, linear damage begins to evolve in the materials once the initial damage reaches its critical value, that is, the criterion denoting onset of the initial damage. The response of the model materials during failure is mainly reflected by using cohesive elements, therefore, quite a large stiffness is used to allow complete transmission of force from solid elements in the elastic stage. The response of model materials after reaching peak stress is simulated by utilising the descending segment of the stress–strain relationship for these cohesive elements.

**Figure 5:**
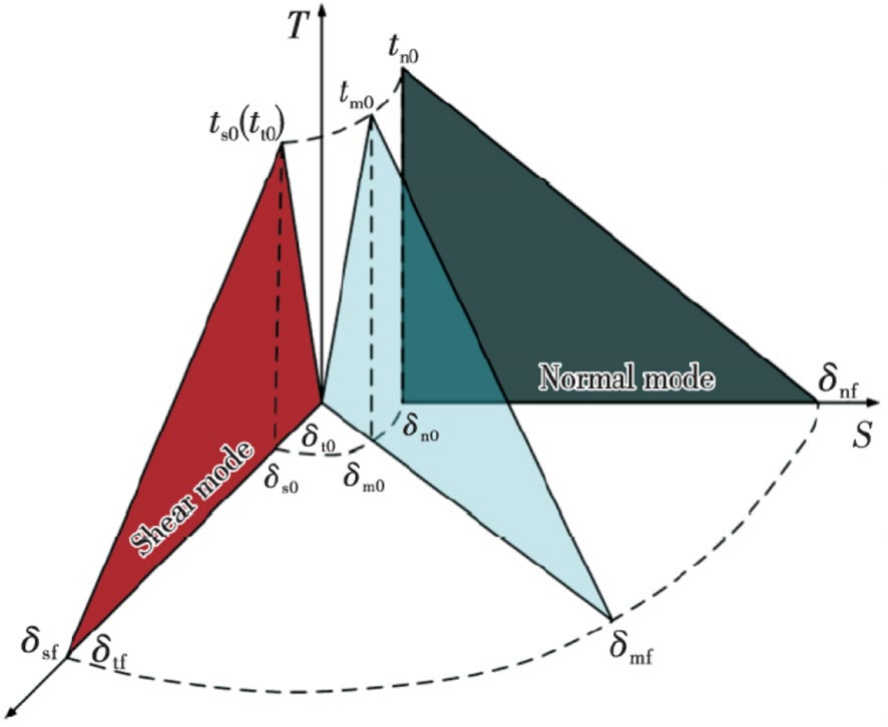
Mixed-mode cohesive traction response.

To explore the combined cracking, the quadratic normal stress criterion is applied (Eq. (1)); the CZM under bilinear combined cracking is shown in Fig. 5^30^, where, “〈〉”, are Macaulay brackets, means that the function has a value of zero when the independent variable is negative; the function equals the independent variable when the independent variable is positive. Macaulay brackets indicates that the initial damage does not occur in a purely compressive state of stress. The vector *τ* of the normal traction stress consists of three components, in which $\tau_{{\text{n}}}$ denotes the component perpendicular to a possibly cracked surface and $\tau_{{\text{s}}}$ and $\tau_{{\text{t}}}$ denote two shear components on the surface where cracks are likely to occur. $\tau_{{\text{n}}}^{0}$, $\tau_{{\text{s}}}^{0}$, and $\tau_{{\text{t}}}^{0}$ represent the peak normal stresses. The onset of damage occurs at *f* = 1.

3 $$f = \left\{ {\frac{{\left\langle {\tau_{n} } \right\rangle }}{{\tau_{n}^{0} }}} \right\}^{2} + \left\{ {\frac{{\tau_{s} }}{{\tau_{s}^{0} }}} \right\}^{2} + \left\{ {\frac{{\tau_{t}^{2} }}{{\tau_{t}^{0} }}} \right\}^{2}$$

The level of damage in a material is characterised by introducing damage variable *D*, which can be defined in terms of displacement and energy. The fracture energy, that is, the energy *G*_f_ per unit area to be absorbed in crack initiation, is used to define the damage variable. Equation (3) shows the bilinear damage variable defined in terms of energy:

4 $$D = \frac{{2G_{f} /T_{0} (\delta_{\max } - \delta_{0} )}}{{\delta_{\max } (2G_{f} /T_{0} - \delta_{0} )}}$$

where, $\delta_{\max }$, $\delta_{0}$, and $T_{0}$ denote the maximum displacement during loading, the displacement corresponding to the onset of damage, and the critical stress under that initial damage, respectively. The Drucker-Prager model is used for solid elements of rocks and the calculation parameters are listed in Table 1. The model parameters are the same as those of physical experiments, and some of them are obtained by repeated trial calculation. The rate-dependence of rock is considered in solid elements and the relationship between the strength and strain rate is given by^31^:

5 $$\sigma_{d} = 0.4\sigma_{cd} (\dot{\varepsilon })^{1/3}$$

where, $\sigma_{{\text{d}}}$, $\sigma_{{{\text{cd}}}}$, and $\dot{\varepsilon }$ refer to the dynamic strength, static strength, and strain rate, respectively.

**Table 1:** CZM model parameter.

Material	Density/kg·m^-3^	Young’s modulus/GPa	Poisson’s ratio	Internal friction angle	Tensile strength/MPa	Shear strength/MPa	Fracture energy
Experimental material	1800	2.03	0.16	35*	/	/	/
Cohesive	/	/	/	/	0.16	0.64	2*
Anchor bolt	3000	76	0.34	/	/	/	/

“*”Represent repeated trial calculation value.

### Simulation of crack propagation

**Figure 6:**
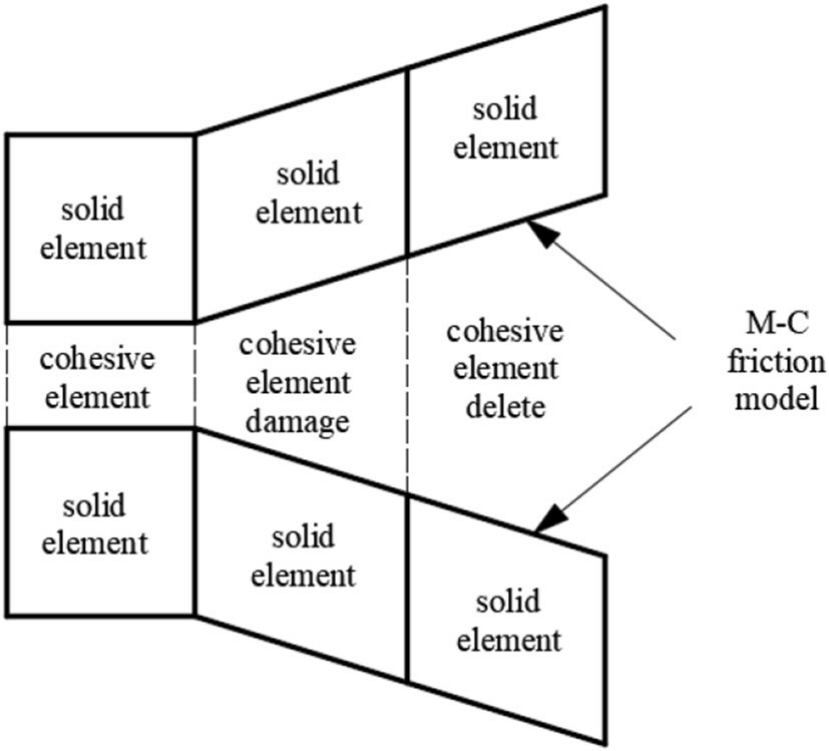
Crack propagation mode.

The solid element mesh is partitioned by ABAQUS; thereafter, zero-thickness cohesive elements are inserted between blocks of all solid elements comprising the mesh by Python, as shown in Fig. 6. During calculation, the elements are deleted when the damage of cohesive elements reaches 1, which means that materials are fractured to form cracks. After the crack surfaces are formed, the interaction between crack surfaces also plays an important role in the subsequent crack propagation. To prevent the interactive invasion of crack surfaces, a hard contact was automatically used on crack surfaces after cohesive elements are deleted. The Mohr–Coulomb friction model was applied as the friction criterion, with coefficient of friction $\mu { = }\tan \varphi$.

### Verification of model accuracy

To test the rationality of the established model and the reliability of results, the accuracy of the model was validated by comparing the time history curves of radial stress on elements at the blast source and the distribution of cracking at failure in the rock surrounding a cavern according to the related test results.

**Figure 7:**
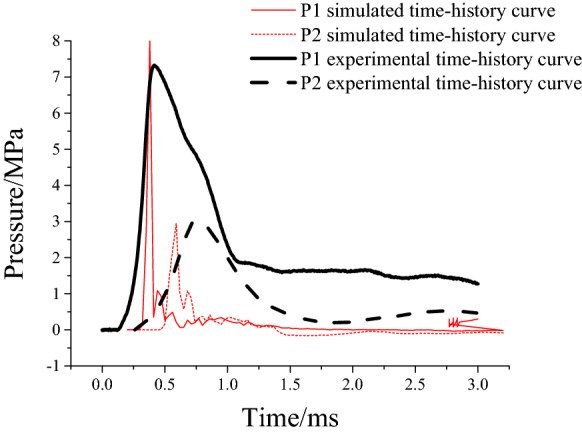
Comparison of simulated and measured compressive stress time-history curves.

**Figure 8:**
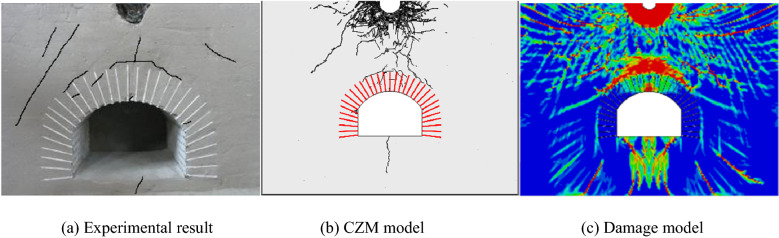
Comparison of simulated damage, simulated crack, and test crack.

1. A comparison was made by selecting time history curves of pressure at two points P1 and P2 at the same positions in the model test, as shown in Fig. 7; the simulation curves obtained through CZM undergo a shorter duration of action relative to that experimentally, mainly because materials are idealised during numerical analysis; however, a certain discrepancy arises in cement mortars used experimentally, which increases the duration of action of stress waves and reduces their peak intensity. The CZN model is actually the coupling of finite elements and discrete elements. The stress immediately decreases after the break of the monitoring point. Overall, the peak stress in the simulation result approximates to that measured experimentally, and the simulated curves are of a similar shape to their experimental counterparts, suggesting that the attenuation law of stress wave intensity is the same as the physical experiment. The physical experiment also monitored that the vault acceleration of the cavern was 1999.8 m/s^2^, while the vault acceleration in the numerical model was 1722.3 m/s^2^. It is appreciable to observe that the value of vault acceleration in the numerical model is akin to the vault acceleration in the experimental monitoring value, which backs the accuracy of the numerical model.
2. The accuracy of crack propagation simulated through the model was verified by comparing the distribution of cracks. Additionally, the physical test was assessed using the continuum damage model and the calculated result is shown in Fig. 8c, in which the red zone corresponds to the damage zone, approximating to areas suffering formation of cracks. Figure 8a, b separately show the result obtained through the physical test and that calculated using the CZM. As shown in the figures, black parts represent the main cracks formed after blasting. By comparing the distributions of cracking across the three results, it can be found that both the damage model and CZM reflect that the most damaged zones around the anchored cavern are mainly found at the boundary and middle part of the anchored zone of the vault as well as the floor. The damage model can reveal those zones where fractures probably appear while it fails to describe the position and path of propagation of such fractures. The CZM can show the distribution and propagation of cracks, which are consistent with distributions obtained through physical testing. A broken zone is formed near the blast source, at the outer surface of which, radial cracks appear. Radial cracks generated due to blasting are deflected to the free faces and the cavern due to the presence of free faces of the top of the cavern. Vertical cracks extending to the blast source are generated at the top of the anchored zone of the cavern and cracks are formed at the boundary of the anchored zone; in addition, some fine cracks appear in the anchored zone and vertical cracks are found at the top of the cavern, arch springings, and the floor.

Above all, the reliability of the calculation model and its superiority relative to the damage model are validated by comparing the time history curves of compressive stress and the distribution of cracks obtained through numerical analysis and model testing.

## Analysis of crack propagation

### Crack propagation process in an anchored cavern with no pre-fabricated fractures

**Figure 9:**
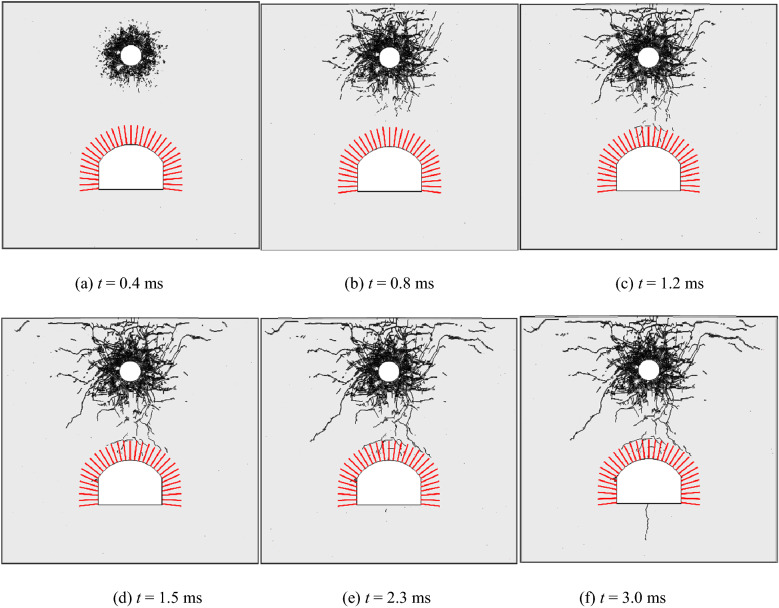
Crack propagation process of anchorage cavity.

The propagation of cracks in the rock surrounding an anchored cavern without pre-fabricated fractures was analysed: Fig. 9 shows the crack propagation pattern under top explosion. Broken zones are formed around the blast source under the huge compressive stress generated by shock waves. The energy generated due to blasting is largely consumed in this stage. On this condition, the peak stress drops to below the compressive strength of the rock while it remains above the tensile strength of the rock, thus generating radial tensile cracks. The stress waves are reflected as tensile waves when propagating to the free face in the top, thus inducing spalling. As stress waves propagate to the anchored zone, fine vertical cracks occur at the ends of the bolts at the vault. In the case that stress waves are reflected as tensile waves on the free face of the vault after passing though the anchored zone, only uncoalesced fine cracks are formed in the anchored zone due to the reinforcement effect of bolts. After the reflected tensile waves propagate beyond the anchored zone, the tensile strength of rock decreases without the confining effect of the bolts beyond the anchored zone, therefore, the coalesced tensile cracks occur at the boundary between the anchored zone and the unanchored zone. The reflected tensile waves at the free faces on the top of the model and in the cavern are mutually superimposed on the stress field at the tip of the radial fractures and therefore the radial fractures separately extend to the free faces forming the top and the cavern. As stress waves pass through the cavern, vertical tensile cracks are generated at the arch springings and the middle part of the floor.

### Crack propagation in an anchored cavern with pre-fabricated fractures

**Figure 10:**
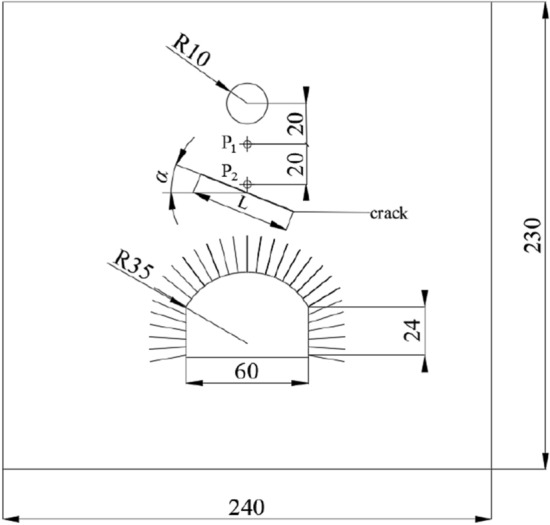
Fracture setting diagram (unit: cm).

Only a single fracture was established to analyse its influence on the distribution of cracks in the rock surrounding this anchored cavern. The pre-fabricated fracture, appearing as a long, narrow ellipse, was classified as an open fracture, with a short-axis length of 0.5 mm. This corresponds to an open fracture with a spacing of 5 mm in the actual cavern, showing the length of 30 cm and a skin friction coefficient of the fracture of $\mu { = }\tan \varphi$. The fracture is set beyond the anchored zone, being 39 cm from the surface of vault, with the dip angles $\alpha$ of 0°, 30°, 45°, 60°, and 90°, as shown in Fig. 10.

Under blast load, two forces are found at the tip of the fracture: the shear stress formed by stress waves after passing through the tip of the fracture and the tensile stress formed at the tip of the fracture. The tip of the fracture constantly advances under the effect of the two forces. The fracture can be classified as exhibiting three modes: open cracks (mode I), sliding mode crack (mode II), and tearing mode cracks (mode III). The last two fracture modes are driven by shear force, so they are also indicative of shear failure. When exploring the mechanism of formation of cracks, many scholars infer the fracture mode of materials according to the stress state in various elements. The numerical model which analyses the causes of cracks based on the proportions of different fracture energies during the failure of cohesive elements can more provide better assessments of the mode of propagation of cracks, to provide better guidance for reinforcing the rock. *R*_*a*_ is defined as the proportion of the shear fracture energy in the total fracture energy when cohesive elements start to be fractured:

6 $$R_{a} = 1 - \frac{{G_{n} }}{{G_{T} }}$$

where, $G_{{\text{n}}}$ denotes the tensile fracture energy; $G_{{\text{T}}} { = }G_{{\text{s}}} + G_{{\text{t}}}$, in which $G_{{\text{s}}}$ and $G_{{\text{t}}}$ separately refer to the shear fracture energy along with two conjugate directions perpendicular to the tensile stress.

**Figure 11:**
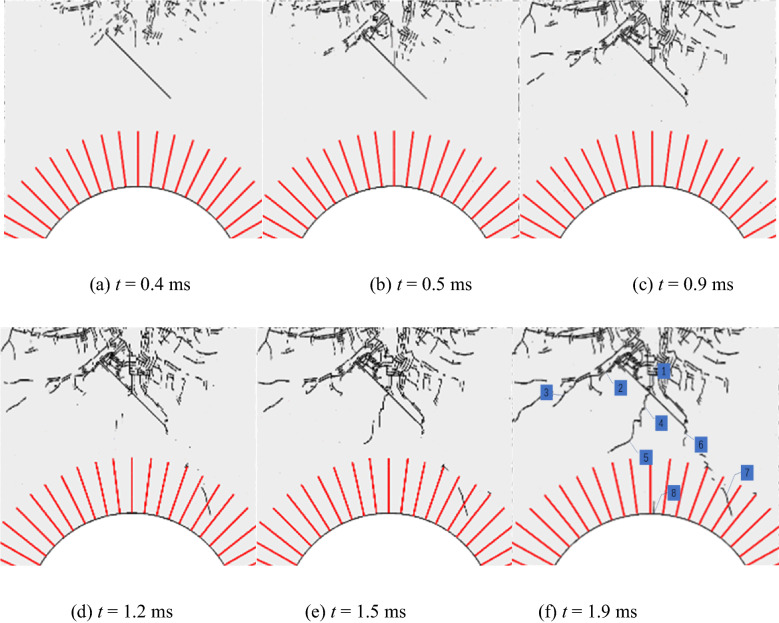
Crack propagation process.

**Figure 12:**
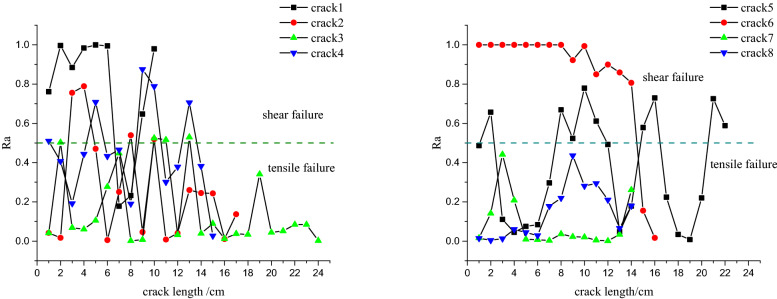
Shear fracture energy ratio.

The mode of propagation of cracks in surrounding rocks of the anchored cavern with a pre-fabricated fracture with a dip angle of 45° was analysed. Figure 11 shows the crack formation and propagation process in surrounding rocks of the anchored cavern and Fig. 12 shows the fracture mode. Cracks are closed under the effect of compression, and the vertical compressive shear crack 1 is first formed from the upper part of the fracture; moreover, the tensile cracks parallel to the fracture surface are formed due to reflected tensile waves on the upper surface of the fracture, and shear cracks parallel to the direction of propagation of stress waves are formed due to closure of the fracture under compression. At the upper tip of the fracture, there are not only cracks coalescing to the fracture zone formed due to blasting but wing crack 2 extends away from the blast source, with a propagation angle of 90°. The wing crack formed at the upper tip of the fracture mainly appears as a tensile fracture; moreover, some elements with *R*_a_ > 0.5 are subjected to tensile-shear fracture. This indicates that the tensile stress and shear stress synchronously influence the propagation of the crack tip and the both interact (nevertheless, tensile stress is taken as the main driving force). Crack 6 formed at the lower tip of the fracture is mainly manifest as a shear crack. When stress waves are applied to the fracture, it is dislocated, therefore, the vicinity of the lower tip of the fracture is subjected to shear failure to form short sliding-mode cracks. The tensile waves reflected from the free face of the vault are applied at the tip of the wing cracks to form a tensile fracture, which propagates towards the vault. In addition, the reflected tensile waves continue to propagate to form tensile crack 3 at the end of the wing crack at the upper tip. Cracks 4 and 5 formed under the interaction of tensile and shear failures are formed between the pre-fabricated fracture and the vault after stress waves pass through the fracture. Tensile cracks 7 and 8 are formed in the middle and right-hand side of the anchored zone of the vault.

According to Fig. 11, the cracks around the anchored zone in the anchored cavern with a pre-fabricated fracture mainly appear as tensile cracks under blast load, therefore, measures for preventing tensile failure are mainly taken in the vicinity of the anchored zone during its reinforcement. The cracks propagating from the middle and lower tip of the pre-fabricated fracture are shown as shear cracks and therefore reinforcement measures for their prevention should also be taken for the pre-existing fracture in the cavern.

**Figure 13:**
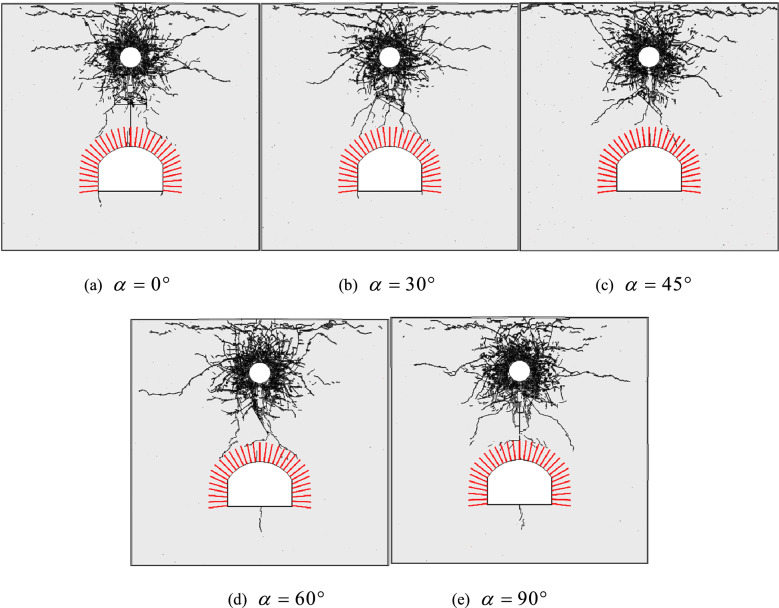
The crack distribution around an anchored cavity with cracks at different dip angles.

Figure 13 shows the distribution of cracks in surrounding rocks of the anchored cavern when a fracture with different dip angles was pre-fabricated in the top of the cavern. When the dip angle of the pre-fabricated fracture is 0°, the wing cracks coalesce with the tensile cracks at the boundary of the anchored zone and vertical cracks are generated along the line connecting the centre of the cracks and the centre of the vault. Moreover, the presence of the fracture changes the direction of propagation of stress waves and attenuates the energy generated by stress waves. Therefore, no reflected tensile cracks are formed in the middle part of the boundary of the anchored zone of the vault while cracks are found only in the two sides. Within the anchored zone of the vault, vertical cracks are generated only at the top while compressive shear cracks appear in corners of the two side walls. As the dip angle of the pre-fabricated fracture increases, the cracks at the boundary of the anchored zone in the left-hand end far from the fracture are gradually reduced and deflected away from the cavern; however, the opposite trend is seen in the right-hand end of the anchored zone. At a dip angle of the pre-fabricated fracture of 45°, the wing cracks at the left-hand end of the fracture do not coalesce to the boundary of the anchored zone while cracks occur in the right half of the anchored zone of the vault. No cracks are formed in the arch foot and floor, which implies that the presence of the pre-fabricated fracture attenuates stress waves and the generation of numerous cracks above the fracture absorbs some of the energy generated by stress waves. When the dip angle of the fracture exceeds 45°, the cracks formed at the left-hand end of the fracture coalesce with those at the boundary of the anchored zone. Owing to the right-hand end of the fracture being close to the anchored zone of the cavern, wing cracks coalesce with the reflected cracks at the boundary of the anchored zone and vertical cracks are also present in the middle of the floor. When the pre-fabricated fracture is vertical, vertical cracks parallel to the fracture are formed from the upper end of the cracks. The distribution of cracks around the cavern is similar to that in the anchored cavern with no pre-fabricated fracture. This is because the fracture is parallel to the direction of propagation of the stress waves and exerts no significant influence on the propagation of stress waves.

## Analysis of the dynamic response of an anchored cavern

### Analysis of the vault displacement in an anchored cavern

**Figure 14:**
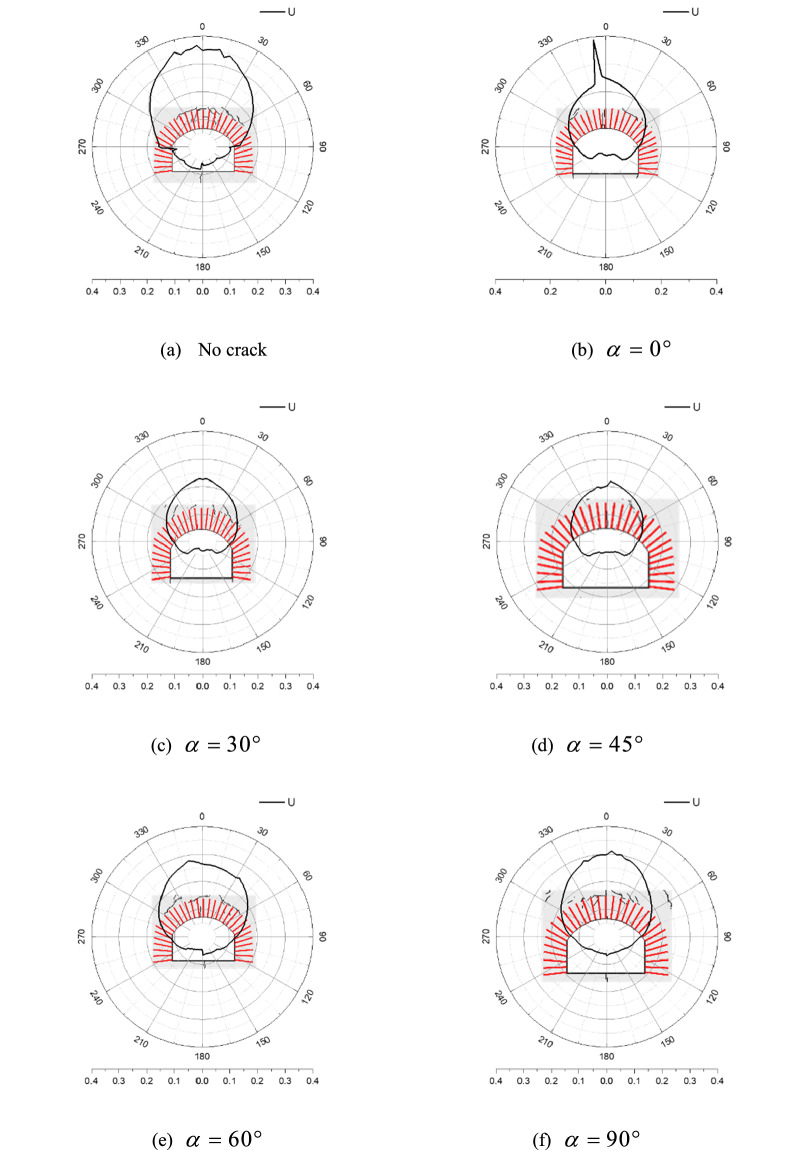
Cavern peak displacement distribution.

Figure 14 shows the distribution of peak displacements in the rock surrounding an anchored cavern, in which 0° and 180° separately correspond to the centres of the vault and floor while 90° and 270° separately correspond to the arch springings. The peak displacement of the vault facing the blast effect is the largest, successively followed by that of the side walls and floor. When no fracture or a fracture is pre-fabricated vertically in the vault, the displacement of the floor exceeds that of the side walls while the former is lower than the latter on condition that the pre-fabricated fracture is inclined; moreover, the displacements of the arch springing and side wall in the right-hand side closer to the pre-fabricated fracture are slightly larger than those on the left. The distributions of the peak displacement are smooth and continuous and the zone of abruptly changing displacement corresponds to the surrounding rocks within which cracks are generated. When the surrounding rocks are exfoliated, the displacement in the corresponding areas increases significantly, for example, the zone with abruptly changing displacement on the vault of the cavern having a pre-fabricated fracture with the dip angle of 0°. On condition that many cracks are present at the boundary and the interior of the anchored zone, the displacement of the corresponding surrounding rocks also increases slightly, indicating that the displacement of the surrounding rocks is somewhat related to the displacement of the cracks.

**Figure 15:**
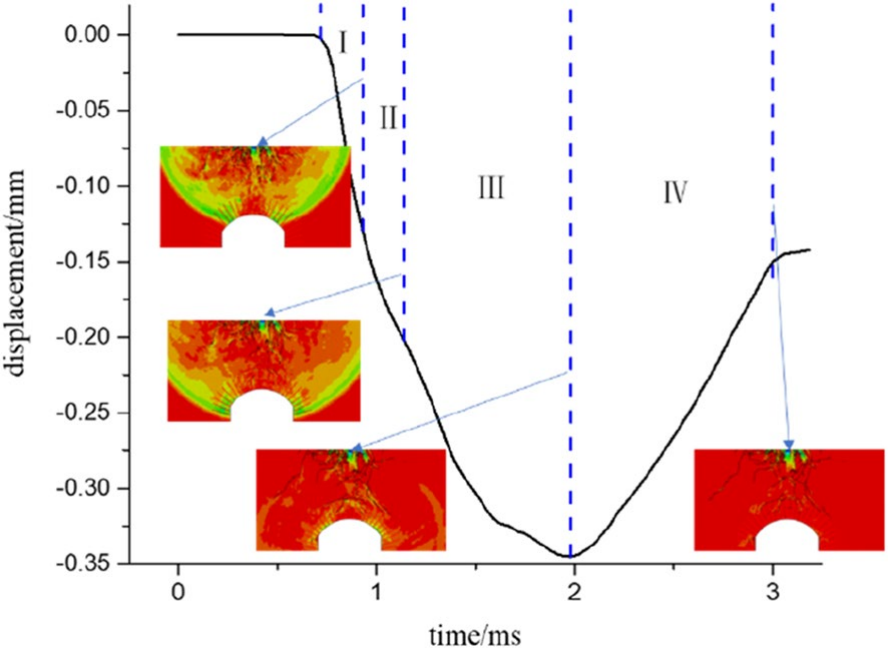
Displacement of the vault of an unfractured cavern.

**Figure 16:**
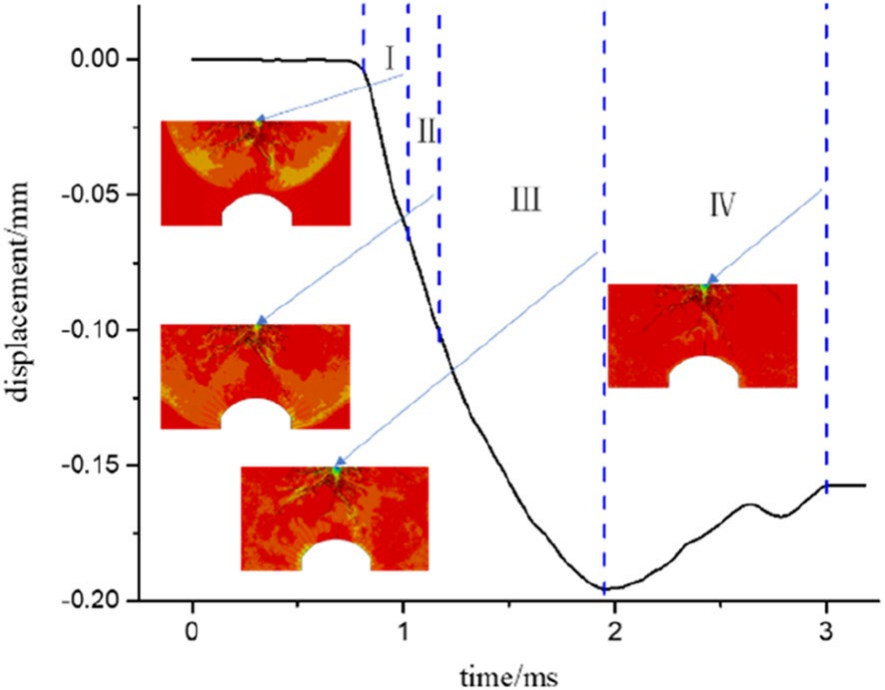
Displacement of the vault of a cavern with a crack.

Figures 15 and 16 separately show the displacements of the vault of an anchored cavern without a fracture and with a pre-fabricated fracture at a dip angle of 45°. A negative represents a downwards displacement. The results obtained experimentally and by numerical calculation reveal that the dynamic response of the anchored cavern ends 3 ms after blasting and thus the displacement at 3 ms is selected as the residual displacement. The vault displacement is divided into four phases: in phase I, the vault is deformed under the effect of stress waves and no cracks occur in the anchored zone of the vault. The deformation is manifest as elasto-plastic deformation of the upper part of the cavern. The deformation is insignificant in this phase. In phase II, cracks are found in the anchored zone; however, there are only a small number of cracks therein and the vault displacement is low. In phase III, cracks occur outside the anchored zone. After the reflected waves from the free face of the vault pass out of the anchored zone, numerous cracks are formed at the boundary of the anchored zone. The vault displacement occurs mainly in this phase. In phase IV, the elastic displacement recovers. The elastic deformation in rocks and that of bolts for the anchored zone gradually recover and therefore the vault will be displaced upwards to some extent. As shown in Figs. 15 and 16, the vault displacement of the anchored cavern with a pre-fabricated fracture starts to change later because the presence of the fracture hinders the propagation of stress waves. Therefore, the vault displacement begins to vary later; moreover, the presence of the fracture attenuates stress waves and thus the displacement of the anchored cavern with a pre-fabricated fracture is lower than that without a fracture in phase I. As for the anchored cavern with a pre-fabricated fracture, the cracks around the fracture continue to propagate under the effect of reflected waves in phase III; in addition, the recovered displacement is lower than that of the anchored cavern without the fracture in phase IV. The reason for this is that the presence of many cracks around the pre-fabricated fracture in the vault weakens the rock in the upper part, leading to a large residual displacement. It can be seen from Table 2 that the peak displacements all decrease slightly when a fracture is pre-fabricated in the vault compared with those of the anchored cavern without a fracture, in which the peak displacement is the lowest when the dip angle of the pre-fabricated fracture is 45°; the residual displacements of the anchored cavern with a pre-fabricated fracture are all larger than those without a fracture, except when the dip angle of the fracture is 0°, in which the residual displacement reaches its maximum at a dip angle of 90°.

**Table 2:** Vault displacement.

Dip angle	No crack	0°	30°	45°	60°	90°
Peak displacement	0.347	0.233	0.207	0.195	0.260	0.295
Residual displacement	0.149	0.137	0.156	0.157	0.151	0.235

### Analysis of the particle velocity in an anchored cavern

**Figure 17:**
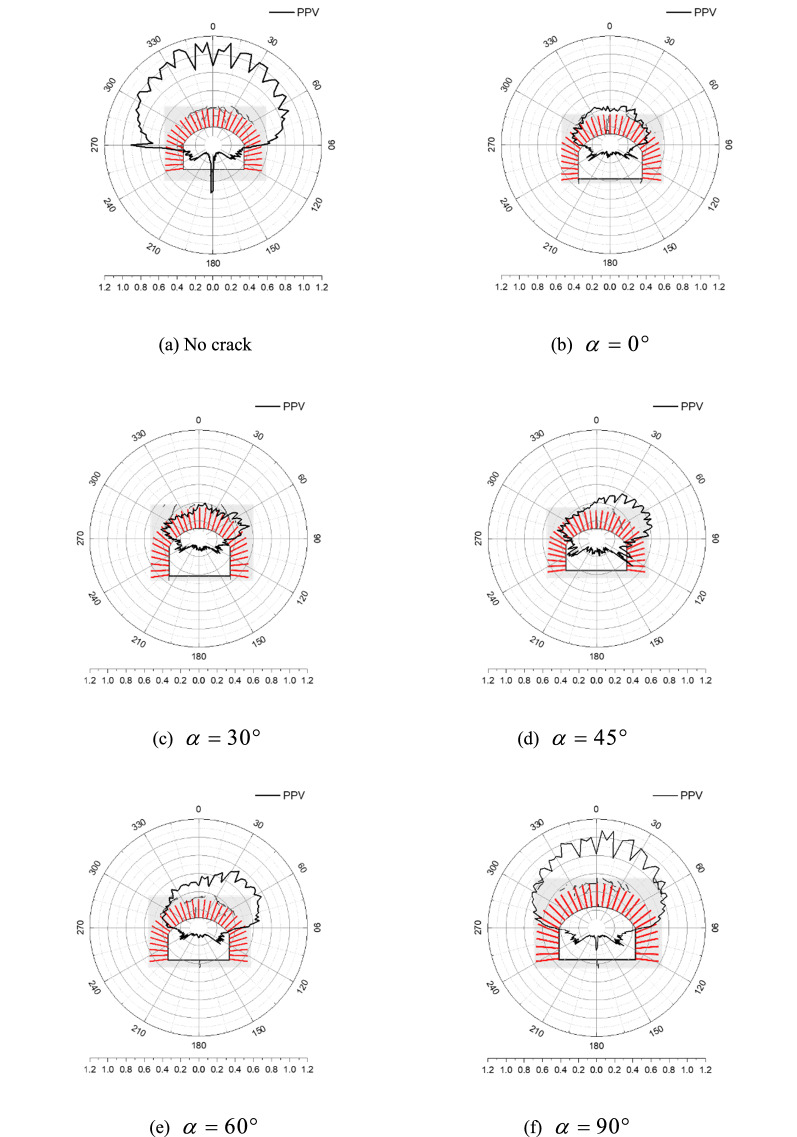
PPV distribution in caverns.

Figure 17 shows the distribution of PPVs around an anchored cavern, in which 0° and 180° separately correspond to the centres of the vault and floor while 90° and 270° separately correspond to arch springings. The distribution of the PPV appears serrated, positions with a low PPV correspond to those reinforced by bolts while the zones of lower reinforcement effect between adjacent bolts have a larger PPV, indicating that bolts reduce the PPV around parts of the cavern. For an anchored cavern without a pre-fabricated fracture, the PPVs are quasi-symmetrically distributed, in which the PPV is largest in the vault, successively followed by that in the side walls and floor: however, due to the differences in the distribution of cracks in surrounding rocks, the PPV abruptly changes in local zones where cracks appear. For example, spalling or radial cracks are formed in the left-hand arch springing and the centre of the floor, where the PPV increases the most. The PPV decreases significantly when a pre-fabricated fracture is present in the upper part of the vault. With increasing dip angle of the pre-fabricated fracture, the PPV distribution in the surrounding rocks becomes asymmetric: the PPV in the anchored zone in the right-hand end closer to the pre-fabricated fracture is larger owing to more cracks being generated in this zone. Above all, the PPV in surrounding rocks is the lowest when a horizontal fracture is pre-fabricated in the vault; the PPV in surrounding rocks for a dip angle of the pre-fabricated fracture of 90° approximates to that of the anchored cavern without such a fracture; however, the PPV is also slightly lower than that of the anchored cavern without the fracture due to energy absorption along the pre-fabricated fracture.

## Discussion

According to analyses of the crack propagation and dynamic response of the anchored cavern, it can be found that the CZM can integrate analysis of crack propagation at the mesoscopic scale with the dynamic response at the macroscopic scale. Under blast load, when joint planes such as fractures are present on the top of the cavern, they give rise to two effects on the stability of the cavern: on the one hand, the fractures block the propagation of stress waves, especially open fractures. Numerous propagating fractures are present in the vicinity of pre-existing fractures, absorbing much of the energy associated with the input stress waves; on the other hand, fractures weaken the rock in the upper part, resulting in increasing residual displacement of the vault.

According to the distribution of PPV in surrounding rocks of the cavern, it can be found that bolts decrease the PPV and act to restrain crack propagation. The existing safety criterion applied during blasting vibration is mainly discussed in relation to unsupported caverns. According to Fig. 17, cracks have been formed in the floor of the cavern that is unreinforced by bolts at a low PPV; by contrast, a larger PPV is required to form cracks in the anchored zone. The speed scale factor of the model is 0.3. By transforming the vibration velocity into that during practical engineering operations, cracks occur in the unanchored zone at a PPV of 60 cm/s, which is similar to those found elsewhere^32–35^ However, the PPV must reach about 120 cm/s (about three times that in the unanchored zone) so that cracks can be generated in the reinforced (anchored) zone, therefore, it is necessary to explore the safe vibration velocity during blasting in an anchored cavern.

## Conclusion

Based on a CZM, the crack distribution and propagation in an anchored cavern without and with a pre-fabricated fracture at different dip angles under blast load were simulated by globally embedding cohesive elements with zero thickness. In this way, the feasibility of using the CZM to simulate the fracture and failure of caverns was validated; moreover, a comparison was undertaken between the influences of the dip angles of the pre-fabricated fracture in the vault on the crack propagation and dynamic response. The following conclusions are drawn:

1. The CZM can simulate the cracking and propagation of cracks by embedding cohesive elements with zero thickness between solid elements. By comparing with the physical test, the CZM simulates the distribution of cracks in an anchored cavern without a pre-fabricated fracture. Additionally, the feasibility of the model when used to simulate crack propagation in an anchored cavern is verified.
2. Under top explosion, cracks around the anchored cavern without a pre-fabricated fracture mainly occur at the boundary of the anchored zone of the vault, arch springing, and the middle of the floor. When a pre-fabricated fracture is present in the vault, numerous reflected tensile cracks and vertical shear cracks are formed above the fracture due to the blocking effect of the fracture on stress waves. In addition, wing cracks are formed at the tip of the fracture; the number and extent of reflected tensile cracks at the boundary of the anchored zone of the vault decrease significantly, decreasing at first, then increasing with increasing dip angle of the pre-fabricated fracture. There are fewest cracks at the boundary of the anchored zone at a dip angle of 45°. Tensile cracks are mainly found around the anchored zone while shear cracks also appear in the vicinity of the pre-fabricated fracture. When reinforcing surrounding rocks, measures should be taken in the vicinity of the anchored zone to prevent tensile failure; by contrast, the reinforcement measures used for preventing shear failure should be also taken as for pre-existing fractures in the cavern.
3. Under blast load, the vault displacement of the anchored cavern is mainly attributed to tensile cracks formed in the anchored zone due to the reflected tensile waves on the free face of the vault. When a fracture is pre-fabricated in the upper part of the vault, the blocking effect of the fracture on stress waves leads to the reduction of the peak displacement of the vault; moreover, the presence of the fracture weakens the rock in the upper part, resulting in greater residual displacement.
4. The distribution of PPV in the anchored cavern is serrated: the vibration velocity in the anchored zone is lower than that in the unanchored zone. In terms of the overall distribution of PVV, the vault shows the largest PVV, successively followed by the side walls and the floor. In addition, the PVV increases abruptly in positions subjected to spalling and cracking. When a pre-fabricated fracture is present in the upper part of the vault, the PVV decreases significantly; the PVV in the right half of the anchored zone closer to the pre-fabricated fracture is larger than that on the left. The lowest PVV in surrounding rocks occurs when a pre-fabricated fracture is established in the horizontal direction. The PVV enabling formation of cracks in the anchored zone is about three times larger than that in the unanchored zone.
